# Morphological and Genetic Diversity within Salt Tolerance Detection in Eighteen Wheat Genotypes

**DOI:** 10.3390/plants9030287

**Published:** 2020-02-25

**Authors:** Ibrahim Al-Ashkar, Ali Alderfasi, Walid Ben Romdhane, Mahmoud F. Seleiman, Rania A. El-Said, Abdullah Al-Doss

**Affiliations:** 1 Plant Production, College of Food and Agriculture Sciences, King Saud University, Riyadh 11451, Saudi Arabia; aderfasi@gmail.com (A.A.); wromdhane@ksu.edu.sa (W.B.R.); or mahmoud.meleiman@agr.menofia.edu.eg (M.F.S.); aaldoss@ksu.edu.sa (A.A.-D.); 2 Agronomy Department, Faculty of Agriculture, Al-Azhar University, Cairo 11651, Egypt; 3 Department of Crop Sciences, Faculty of Agriculture, Menoufia University, Shibin El-kom 32514, Egypt; 4 Biological and Ecological Department, Faculty of Home Economic, Al-Azhar University, Tanta 31732, Egypt; RaniaEl_said@azhar.edu.eg

**Keywords:** salinity tolerance, genetic diversity, wheat breeding, doubled haploid lines, stepwise regression, SSR markers

## Abstract

Salinity is a major obstacle to wheat production worldwide. Salt-affected soils could be used by improving salt-tolerant genotypes depending upon the genetic variation and salt stress response of adapted and donor wheat germplasm. We used a comprehensive set of morpho-physiological and biochemical parameters and simple sequence repeat (SSR) marker technique with multivariate analysis to accurately demonstrate the phenotypic and genetic variation of 18 wheat genotypes under salinity stress. All genotypes were evaluated without NaCl as a control and with 150 mM NaCl, until the onset of symptoms of death in the sensitive plant (after 43 days of salinity treatment). The results showed that the relative change of the genetic variation was high for all parameters, heritability (>60%), and genetic gain (>20%). Stepwise regression analysis, noting the importance of the root dry matter, relative turgidity, and their respective contributions to the shoot dry matter, indicated their relevance in improving and evaluating the salt-tolerant genotypes of breeding programs. The relative change of the genotypes in terms of the relative turgidity and shoot dry matter during salt stress was verified using clustering methods. For cluster analysis, the genotypes were classified into three groups: tolerant, intermediate, and sensitive, representing five, six, and seven genotypes, respectively. The morphological and genetic distances were significantly correlated based on the Mantel test. Of the 23 SSR markers that showed polymorphism, 17 were associated with almost all examined parameters. Therefore, based on the observed molecular marker-phenotypic trait association, the markers were highly useful in detecting tolerant and sensitive genotypes. Thus, it considers a helpful tool for salt tolerance through marker-assisted selection.

## 1. Introduction

Soil salinization and poor water quality are a worldwide problem in crop production. A high level of salt reduces growth, motivates leaf damage, and ultimately, causes the death of the crop. Therefore, salinization may threaten the sustainability of crop production and affect human life in areas prone to salinity, which incorporates all of today’s irrigated land [1]. In addition, the lack of water in these areas could lead to an increase in the use of brackish water for producing staple crops.

Wheat plants (*Triticum aestivum* L.) are generally sensitive to salinity. It has been demonstrated that the genotypes that are tolerant of salinity can remain alive but the productivity is greatly reduced at a greater level of 120 mM NaCl [2,3]. Salinity tolerance is a polygenic trait, which has a continuous range of variation and is influenced by genetic and environmental factors, hence affects the phenotypic responses of plants under salinity stress [4,5,6]. Despite the existence of several high-yielding wheat varieties, cultivating genotypes provides opportunities for achieving improved salinity tolerance of wheat through breeding. Continuous breeding is necessary to satisfy the demands of the world’s increasing population, environment, changing climate, and pests. However, successful implementation requires donor genotype sources, efficient screening methods using multivariable screening criteria, biotechnology tools, and an accurate and comprehensive understanding of the genetics, physiology, and related sciences of the mechanisms behind salinity tolerance [7].

Plant responses to stress incorporates a set of temporal scales, from in just a few seconds to largely noticed evolutionary processes. We understand at least three clear time scales of plant response to stress, i.e., response of stress, acclimation, and adaptation. The stress response varies with developmental stages and genotype, and the ability to survive under stress conditions is proof of plant tolerance. Salt-tolerance consists of complex responses at the molecular, metabolic, cellular, and physiological levels and many other responses, e.g., mechanisms controlling ion uptake, osmotic regulation, transport, balance, antioxidant metabolism, and hormone metabolism. Moreover, stress signals play significant roles in increasing a plant’s resilience against salinity stress [8,9]. Gupta and Huang [8] highlighted how, despite significant progress in the comprehension of plant stress responses, there are still large gaps in our knowledge concerning the mechanisms behind transmembrane ion transportation, sensors and receptors within signal transportation, molecules in far-reaching signals, and metabolites in the supply of energy. The implementation of a comprehensive and integrated approach is essential for identifying the major routes or processes that control salinity tolerance [8,9,10].

Despite many studies having investigated the screening procedures for the tolerance of wheat to salinity, the replicability of the experiments and consistency of results between laboratories worldwide continue to be a challenge, owing to a lack of a standard growth environment [11,12] and because the salinity tolerance of wheat changes with each growth stage [2]. Many studies have focused on the screening method [13] but few have been large-scale studies [14]. Although salinity tolerance is a polygenic trait, many studies have considered it as a single trait and used visual scoring during the evaluation [11]. Yeo et al. [15] stated that pyramiding of favorable morphological, physiological, and biochemical parameters can increase salinity tolerance. Statistical model that integrates each of the morphological, physiological, and biochemical parameters would be more appropriate when formulating findings regarding sensitive and tolerant genotypes [6]. Therefore, multivariate analysis is instrumental in identifying sources of genetic variation and discriminating their salt tolerance using multiple selection criteria.

In general, the final target for breeding programs is to improve productivity, quantity, and quality under stress. With this aim, methods for detecting the salt tolerance of a large number of genotypes need to be inexpensive, quick, and easily measurable [16,17]. Testing genotypes for salt tolerance under laboratory conditions may be useful, as it will provide more accurate data. However, there is a significant relationship between the resistance to abiotic stresses observed in the field and laboratory [17,18]. Moreover, it is difficult to accurately measure root traits in the field [19]. Morphological measurements often require substantial phenotypic data and the repetition of cropping seasons for screening and evaluation, owing to the variation in environmental conditions from season to season. Variability in agricultural soil can also have adverse effects on field evaluations, which results in an increase in the coefficient of variation, potentially misleading breeders from achieving their goals [20,21].

The genetic identification of wheat cultivars/lines using morphological measurements alone is not enough. Due to advances in molecular genetics, various techniques have recently emerged for assaying genetic variation. DNA markers are powerful tools for assessing genetic variation, which have advantages in that the DNA content of a cell cannot be influenced by the environmental conditions, stages of plant development, or type of organ, which has significant bearing on the reliability of the results [22,23,24].

Molecular marker technologies have been used in genetic studies, molecular-assisted selection (MAS), paternity analysis, quantitative trait loci (QTL) mapping, assessing genetic variability, cultivar identification, phylogenetic relationship analysis, and genetic mapping. Wheat genome have been extensively sequenced and annotated. Lately, many genome-wide association studies (GWAS) have been conducted to focus on wheat agronomic traits and abiotic tolerance for linking phenotypic variation with genetic variation [25,26]. Simple sequence repeat (SSR) markers are among the most important molecular markers. They are multi-allele, highly informative, accurate, and impartial. SSR markers occur with a high relative abundance and locus specificity and are less time-consuming than other markers. They are distributed widely across the genome, are repeatable across environments, and above all, they are co-dominant and high-throughput in nature [27,28,29]. This all makes SSR markers a good option for genetic diversity studies concerning cultivars and lines of wheat, due to their importance and high yield demand. They are very advantageous for various applications in genetic research and breeding.

Almanza-Pinzon et al. [30] found that the tested primers were highly informative and capable of distinguishing between wheat genotypes for salt tolerance, as they complied with those obtained using different SSR primers [29,31,32]. The identification of Nax1 gene was performed using the SSR markers Xgwm312 and Xwmc170, contributing to the detection of the tolerance genes, as a result of their association with specific DNA fragments that are closely linked to specific genes affecting the salt tolerance of wheat located on the long arm of chromosome 2A [33,34]. Therefore, SSR analysis can play a significant role in identifying the main genes linked to salt tolerance, which can help plant breeders to determine the wheat genotypes with a high salt tolerance and those usable as novel genetic resources for the population structure in breeding programs.

Naturally, the performance of the morphological, physiological, and biochemical parameters will vary among wheat genotypes, with a genotype being superior in at least one trait and inferior in other traits under salt stress. In this study, we aimed to explore these differences to examine the possibility of effectively using these parameters as reliable screening criteria for the evaluation of genotypes under salinity conditions and to validate these screening criteria using different SSR markers linked to salt tolerance. Our goal was also to estimate the genetic variability among eighteen tested genotypes in order to select genotypes of higher salt-tolerance. 

## 2. Materials and Methods 

### 2.1. Plant Material

Eighteen wheat genotypes were tested for salinity tolerance (Table S1). Fifteen doubled haploid lines (DHLs) were selected based on their good grain yield performance. The DHLs were obtained from the Agronomy Department, Faculty of Agriculture, Al-Azhar University, Nasr City, Cairo, Egypt and published by El-Hennawy et al. [35]. The other three check varieties were obtained from the Agricultural Research Center, Egypt and included Sakha-93 (salt-tolerant), Giza-168 (salt-intermediate), and Gemmeiza-9 (salt-sensitive), as described by El-Hendawy et al. [2] and Abdelsalam [36] carried out in Greenhouse and Biotechnology Laboratory, Plant Production Department, College of Food and Agriculture Sciences, King Saud University, Riyadh, Saudi Arabia. 

### 2.2. Screening for Salinity Tolerance at the Vegetative Stage

Grains of the 18 wheat genotypes were incubated at 10 °C for 3 days to completely eliminate any seed dormancy. Sixty grains from each genotype were germinated in petri dishes for 5 days at 25 °C and then transferred into seedling trays of washed sand. After one week, the seedlings were allowed to grow on quarter-strength Hoagland’s hydroponic nutrient solution (pH 5.5–6.0) [37]. Then, 150 mM NaCl was added to the nutrient solution. In addition, control plants were grown under the same conditions without NaCl. All experiments were conducted in a greenhouse at temperatures of 24–29 °C during the day and 18–22 °C during the night, a photoperiod cycle of nearly 12 h of light, and a light intensity of nearly 60 μmol m^−2^ s^−1^. The entire experiment was carried out in a randomized block factorial design and replicated three times per factor and genotype (3 replications with 20 plants each). After 43 days of salinity treatment, at the first instance of symptoms of death, the plants were harvested. The mean value of five plants and/or samples of uniform growth per factor, genotype, and replicate were used to measure the selected growth traits. Relative trait changes (RTC) were calculated as (C−D)/C for all parameters measured.

#### Measurement of Growth Parameters 

Shoot traits, such as shoot length (SL; cm) and shoot dry weight (SDM; g) and root traits, such as root length (RL; cm), number of roots (RN; seminal and nodal roots together), and root dry weight (RDM; g), were estimated at the time of harvest (at 55 days after sowing). The samples for the SDM and RDM were dried in the oven at 70 °C for 48 h.

#### Measurement of Physiological Parameters

The leaf water status was identified through various measurements, including the relative water content (RWC), relative turgidity (RT), and water deficit (WD), as described by Grzesiak et al. [17] and Weatherley [38]. Leaf samples approximately 5-cm-long were collected from fresh leaves and then weighed. The fresh weight (FW) of the samples was recorded before they were drenched for 4 h in 100 mL of distilled water. The turgid weight (TW) of the leaves was then recorded. After that, the same samples were dried completely in an oven at 70 °C for 48 h to reach the dry weight (DW). The parameters were calculated as follows:

WC = FW − DW/FW

RT = FW − DW/TW − DW

WD = 100 − RT

For the total chlorophyll content (ChL), a leaf sample (0.1 g) was collected and placed in 3 mL of methanol. The ChL was identified as follows: total chlorophyll = 25.8 × A650 + 4.0 × A665. The absorbance (A) was measured at 650 and 665 nm using a spectrophotometer (Ultrospec 2100 Pro, MA, USA). The ChL was then converted into micrograms of chlorophyll per gram of leaf tissue. The formula: ChL = (μg chlorophyll/mL methanol) × 3 mL methanol/(g tissue) [39] was used to calculate the ChL.

For the membrane stability index (MSI), a leaf sample (0.1 g) was collected and placed in 10 mL of distilled water. Samples were retained for 30 min at 40 °C, then the conductivity (EC1) was measured using a conductivity meter. After that, the same leaf samples were placed in a boiling water bath (100 °C) for 15 min, and the conductivity (EC2) was measured a second time. The MSI was calculated using the following equation: [1 – EC1/EC2]/100 [40].

#### Extraction and Assay of Antioxidant Enzyme 

For enzyme extractions, fresh leaf samples (0.5 g) were crushed in liquid nitrogen, to grind them into a fine powder, and placed in an ice-bath with 4 mL of a homogenized 50 mM potassium phosphate buffer (pH 7.8), including 1% (w/v) polyvinylpolypyrrolidone. The homogenate was then centrifuged at 14000 rpm for 10 min at 4 °C. This supernatant is used as the rough extract of assays for catalase (CAT; U g^−1^ FW mL^−1^), peroxidase (POD; U g^−1^ FW mL^−1^), and polyphenol oxidase (PPO; U g^−1^ FW mL^−1^), as described by Aebi [41], Chance and Maehly [42], and Duckworth et al. [43], respectively.

### 2.3. DNA Extraction and SSR Analysis

To isolate the DNA, fresh leaf samples (0.5 g) of each genotype were crushed in liquid nitrogen. The genomic DNA of each genotype was extracted using the Wizard Genomic DNA Purification Kit (PROMEGA Corporation Biotechnology, Madison, Wisconsin, USA). A spectrophotometer (Thermo scientific, Wilmington, DE, USA) was used to measure the DNA concentration at 260 nm, and the extract was electrophoresed on 0.8% agarose to ensure the quality. After that, the refined DNA was standardized at 25 ng μL-1, and the final concentration was kept at −20 °C. The 43 SSR markers used in this study were selected from previous studies [30,31,32,44] or the Graingenes website (http://wheat.pw.usda.gov/ggpages/maps.shtml; accessed on 10 November 2019), as they have been linked with salt tolerance in wheat (Table S2). For each 20 μL reaction, the PCR reaction mixture contained 8 μL nuclease-free water, 10 μL 1× GoTaq green master mix (Promega Corporation, Madison, Wisconsin, USA), 0.5 μL primer, and 1.5 μL 25 ng of genomic DNA. The PCR profiles for the SSR analysis were run for 40 cycles of: 94 °C for 1 min, annealing at 51–61 °C (as recommended for each SSR primer) for 1 min, and an extension at 72 °C for 2 min, with a final extension at 72 °C for 10 min. The PCR products were analyzed via capillary electrophoresis using the QI Axcel Advanced system device (Qiagen, Hilden, Germany). 

### 2.4. Statistical Analysis 

Phenotypic Data Analysis: All studied parameters were subjected to an analysis of variance (ANOVA) using SAS software (Version 9.2; SAS Institute, Inc., Cary, North Carolina, USA). The broad sense heritability (h^2^) and genetic gain (GG) were calculated for all studied parameters, as described by Abdolshahi et al. [19]. Stepwise multiple linear regression (SMLR) analysis between all studied parameters was performed using the XLSTAT statistical package (Version 2018; Excel Add-ins soft SARL, New York, NY, USA), with the shoot dry weight (SDM) as the intrigued variable. A matrix to evaluate the pairwise phenotypic dissimilarity between the genotypes was calculated based on the Euclidean distance dissimilarity coefficient; agglomerative hierarchical clustering (AHC) analysis was performed using the Ward’s method in the XLSTAT statistical package (Version 2018; Excel Add-ins soft SARL, New York, NY, USA).

Genotypic Data Analysis: SSR data were scored visually for allele size and presence or absence of each primer. SSR bands were scored as a qualitative character, e.g., present (1) or absent (0), to create a binary matrix. The polymorphic information content (PIC) for each marker was calculated as described by Smith et al. [45]. The discrimination power (Dp) for each marker was calculated as described by Khierallah et al. [23]. To evaluate the genetic diversity, an AMOVA, genetic distance, and Mantel’s test were performed using GenAlEx [46]. A matrix to evaluate the pairwise genetic dissimilarity between the genotypes was calculated based on the Jaccard dissimilarity coefficient; agglomerative hierarchical clustering (AHC) analysis was performed using the unweighted pair group average method (UPGMA) in the XLSTAT statistical package (Version 2018; Excel Add-ins soft SARL, New York, NY, USA).

## 3. Results

### 3.1. Phenotypic Analysis of Salinity Tolerance

#### Analysis of Variance and Mean Performance of the Studied Traits 

The analysis of variance revealed highly significant (*p* < 0.01) differences for the 13 measured traits among the wheat genotypes in the control and salinity treatments. In general, the tolerant check cultivar (Sakha-93) had better mean values for all traits than the sensitive check cultivar (Gemmeiza-9) under salinity treatment, with the moderate check cultivar (Giza-168) midway between them (Table 1). The mean values for most measurements of the 15 DHLs under salinity treatment were near to the mean values of the moderate salinity check cultivar (Giza-168), excluding the RL, CHL, RWC, RT, and WD traits, which were nearer to Sakha-93 or Gemmeiza-9. The maximum values of all traits obtained from the DHLs were higher than the mean values of the tolerant check cultivar (Sakha-93) under salinity treatment, except for the RL and RT. In general, ChL and MSI traits were gradually decreased under salinity and were more visible at the sensitive genotypes when compared to tolerant genotypes (Figure S1). In the control and under salinity condition, the highest values for the coefficient of variation (CV > 20%) were observed for the WD and PPO. In addition, the value for the CV of the POD was higher for the salinity treatment than for the control (Table 1).

#### Relative Change of Measured Traits

The ANOVA showed the highly significant differences between the genotypes as sources of variance, due to the relative change between the control and salinity treatments for all measured traits. The broad sense heritability (h^2^) and genetic gain (GG) showed high values (>60% and >20%, respectively) for all measured traits, which ranged from 79.91% to 97.98% and 44.62% to 288.11%, respectively (Table 2). The relative change of the DHLs compared to the tolerant (Sakha-93) and sensitive check cultivars (Gemmeiza-9) for the studied traits is notable. No DHL had significantly changed for the better compared to the tolerant check cultivar (Sakha-93) for eight traits (RN, RL, RDM, SL, RWC, RT, POD, and CAT). While four DHLs of SDM, two DHLs of MSI, ten DHLs of CHL, two DHLs of WD, and four DHLs of PPO had significantly changed for the better compared to the tolerant check cultivar (Sakha-93). No DHL had significantly changed for the worse compared to the sensitive check cultivar (Gemmeiza-9) for six traits (RDM, SL, MSI, RWC, RT, and WD). While four DHLs of RN, one DHL of RL, one DHL of SDM, three DHLs of CHL, one DHL of POD, one DHL of PPO, and two DHLs of CAT had significantly changed for the worse compared to the sensitive check cultivar (Gemmeiza-9).

#### Identification of Traits Related to Salinity Tolerance

To decipher the tolerance-related traits and the magnitude of their contribution towards tolerance, we made a regression analysis of 12 measured traits as independent variables, with the SDM as the dependent variable, based on the relative change values. The best model regression showed significant associations for six of the measured parameters with the SDM (*p* > 0.05), while also showing that the RN, RL, SL, MSI, PPO, CAT, WD, RDM, and RT contributed towards 0.988 (Table 3). The results of the stepwise regression showed that the RDM and RT were significantly associated with the SDM and were 0.072 and 0.835, respectively, with an R^2^ of 0.907. 

#### Classification of 18 Wheat Genotypes for Salinity Tolerance 

**Figure 1:**
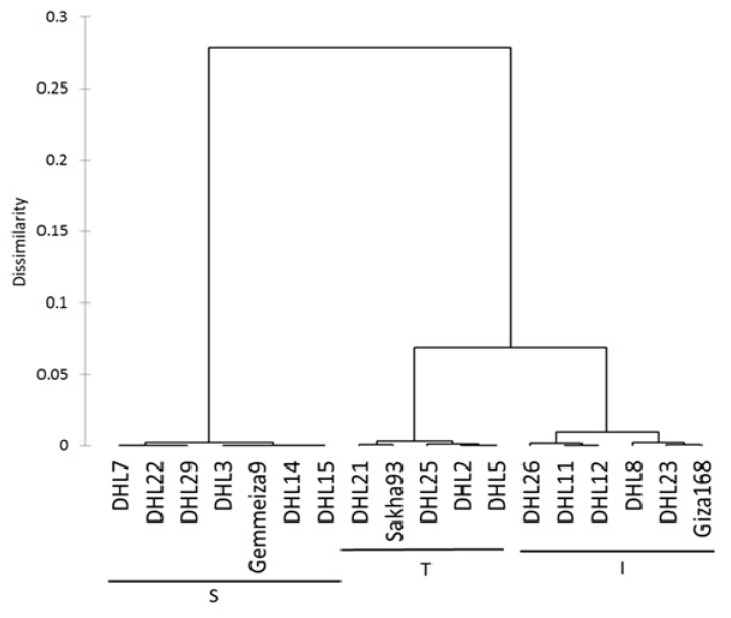
Dendrogram showing clustering of 18 wheat genotypes based on the Euclidean distance of relative change of relative turgidity and shoot dry matter traits. DHL: doubled haploid line, T: tolerant, I: intermediate, and S: sensitive.

The classification was performed based on the relative change calculated for the outputs of the stepwise regression analysis. We decided to use the RT and SDM as parameters in the cluster analysis for the phenotypic classification of the wheat genotypes to confront salinity stress. The dendrogram was generated using the UPGMA method, computed from the two traits (RT and SDM), produced three major clusters, and reflected the distance between the three clusters with similar genotypes (Figure 1). The first cluster was assigned as the tolerant group (T) and included the tolerant salinity check cultivar (Sakha-93), DHL2, DHL5, DHL21, and DHL25. The second cluster was classified as the intermediate group (I), which contained five DHLs (DHL8, DHL11, DHL12, DHL23, and DHL26), as well as the moderate salinity check cultivar (Giza-168). The third group was classified as the sensitive group (S) and contained six DHLs (DHL3, DHL7, DHL14, DHL15, DHL22, and DHL29) and the sensitive check cultivar (Gemmeiza-9).

Further analysis, using one-way ANOVA for the traits (RT and SDM) across three groups, explained that the groups were highly significantly different (*p* < 0.0001) regarding the RT and SDM. Moreover, least squared mean comparisons between the three groups for the RT and SDM traits were significantly different for T compared to I and S, excluding group T, which was insignificantly different for the SDM (Table S3). Group I was significantly different compared to S for the SDM alone. Despite this, the overall pairwise contrasts for the three groups were significantly high in all comparisons, showing the full separation of the groups, which was built upon the quantitative traits.

### 3.2. Microsatellite Genotyping

#### Genetic Diversity of SSR Molecular Markers

Each marker provides a different number of bands that differ from one genotype to another. Out of the 43 different SSR markers tested, only 23 generated polymorphisms among the 18 wheat genotypes, which produced a total of 42 bands. From this, we obtained 35 polymorphic bands, as the percentage of polymorphic loci is 86.23% (Table 4). The number of alleles ranged from one to three, with a mean of 2.33 alleles per locus. The mean value of the PIC of each SSR primer pair set was 0.73, ranging from 0.31 for Barc182 and wmc11 to 0.96 for Cfd60. Most markers had a PIC value greater than 0.50, indicating the ability to analyze the genetic variability of wheat genotypes. The PD ranged between 2.86% to 8.57%, with an average of 4.35%. The groups that showed differences within its members had a higher percentage of polymorphism (100%), with a PIC (polymorphism information content) value of < 0.80 compared to the other markers.

#### Genetic Diversity

Through the assessment of 18 genotypes based on salinity stress responses and their DNA profile, using 42 alleles and 23 SSR markers, the genotypes were separated into three major groups (Figure 1). An analysis of molecular variance (AMOVA) showed significant genetic differences between the three groups (PhiPT = 0.196 at P (rand perm. 999) = 0.001) with 20% and 80% variance among and within the groups, respectively (Table 5). Genetic diversity, with respect to the three salinity tolerance groups (tolerant (T), intermediate (I), and sensitive (S)) showed that the number of polymorphic loci were 33, 32, and 28, respectively, and the percentages of polymorphic loci were 78.14%, 76.05%, and 66.67%, respectively. Group S had fewer numbers of alleles, lower percentages of polymorphic loci, and no unique alleles compared to group T. Based on Shannon’s information index, the donor genotype groups (T and I) showed a higher genetic diversity compared with the genotype group (S), even with smaller sample sizes (Table 6). Similarly, Nei’s genetic distance between genotypes group S is only 0.25, indicating a narrow genetic diversity compared with groups T and I. Among the three groups studied, group (I) showed the highest genetic variability (H = 0.34; I = 0.50; PPL = 76.05%), while group (S) showed the lowest variability (H = 0.25; I = 0.39; PPL = 66.67%) (Table 6). 

#### Clustering and Genetic Relationships between the Wheat Genotypes

**Figure 2:**
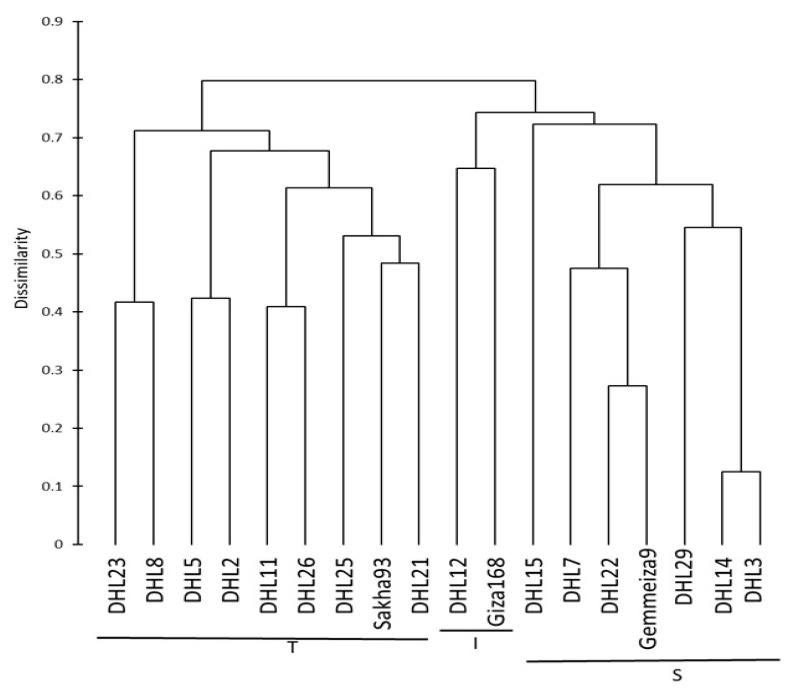
Dendrogram showing clustering of 18 wheat genotypes based on the Jaccard dissimilarity coefficient based on the allelic data of 23 simple sequence repeat (SSR) markers linked to salt-tolerant genes. DHL: doubled haploid line, T: tolerant, I: intermediate, and S: sensitive.

The genetic relationship between the genotypes was evaluated to determine parental genotypes for the breeding program and whether the observed classification of the 18 genotypes, based on their DNA profile, corresponds with their reaction to salinity stress. Conclusions reached from Nei’s genetic distances (Table 6) revealed that the differences among the three groups ranged from 0.089 between groups T and I to 0.138 between groups T and S. These results were confirmed with agglomerative hierarchical clustering (AHC), which showed that the three groups formed three clear clusters (Figure 2). The first cluster contained a mixture of individuals, which included all of the genotypes from group T and four DHLs from group I; the second cluster included only two individuals (Giza-168 and DHL12), while the third cluster was composed entirely with individuals belonging to group S (Figure 1 and Figure 2). Furthermore, the Mantel test was used to calculate the correlation between the genetic and morphological distances (Tables S4 and S5) and showed a highly significant correlation between the genetic and morphological distances (r = 0.514, *p* < 0.0001, and alpha = 0.05). The significance of the correlation between the two matrices (genetic and morphological) may suggest that there is some linkage between the SSR markers used and morphological characters chosen.

#### Identification of Markers Associated with Salinity Tolerance

The genotypic data obtained from the 23 informative SSR loci (out of 42) were used to identify the markers associated with the different parameters and treatments used in the current study. The analysis used stepwise marker regression to identify the markers that have contributed the most to each parameter (quantitative variation). The results show that 17 of the SSR markers are associated with the parameters (R^2^ ranging from 0.077 for wmc60 in the relative change regarding the SL to 0.832 for gwm241 in the salinity treatment regarding the RT and WD), as highlighted by the partial coefficient of determination presented in Table 7. The markers cfd60, gwm241, wmc18, wmc502, gwm160, cfd13, and gwm335 were the highest contributors towards the assessment of some of the tested parameters, as shown by the partial coefficient of determination (R > 0.5). The cumulative coefficient of determination provided a higher contribution to the salinity treatment and/or relative change (R^2^ ranged from 0.435 to 0.931) compared to the control treatment, for all parameters excluding the POD (Table 7). 

## 4. Discussion

### 4.1. Morphological Diversity

Crop breeding programs produce new varieties with the aim of improving the responses of the crop to abiotic and biotic stresses. In creating salt-tolerant cultivars, breeding programs are evaluating diverse genotypes to increase their utility [47]. Salt tolerance is a complicated trait and is caused by a plethora of interrelated mechanisms of morphological, physiological, and biochemical attributes. These attributes are closely linked to the considerable constraints of salinity on plant growth—for instance, osmotic effects, ion toxicity, limitation of CO_2_ gas exchange, water stress, carbon metabolism, metabolic derangements, and oxidative damage. In addition, they operate in coordination to mitigate both the cellular hyperosmolarity and ion imbalance [8,9,10]. The screening of salt-tolerant wheat genotypes is a major step towards selecting the genotypes compatible with the land salinization and poor water quality, which are associated with irrigation.

The increase in land salinization continues to negatively impact yields and has been found to create a harvest index ranging from 0.2 to 0.5, depending upon the duration of treatment and increase in the salt concentration [48]. In addition, low levels of salinity may not reduce yields, although there is a decrease in the leaf numbers, biomass, and leaf area [49]. A major objective of crop breeding programs is the production of new varieties with improved salt tolerance. We evaluated 18 genotypes (15 DHLs and three check cultivars), as well as the morphological, physiological, and biochemical responses after 43 days of salinity treatment, as this signified the onset of the symptoms of death in the sensitive plant. Our results show that significant variances occur among the tested traits of wheat genotypes (Table 1). Under salinity, the SL and SDM decreased in each of the genotypes, with the highest decrease occurring in the sensitive check cultivar (Gemmeiza-9). Similar results were recorded by [50,51]. The decrease in plant growth traits (SL and SDM) has primarily been a result of the inhibition of cell growth and division, owing to Na accumulation [10]. The limitation of leaf growth is the first toxicity phenomenon of salinity, partly because of the decreased hydraulic conductance in plants [52,53,54]. The sensitive check cultivar (Gemmeiza-9) and some salt-sensitive lines exhibited high RN under salinity treatment (150 mM NaCl) compared with the control treatment and tolerant check cultivar (Sakha-93) (Table 1). This may explain that the existence of salinity stress-caused damage to the roots, and plants tend to proliferate roots during periods of increased salt levels to absorb more water and protect the photosynthetic process [9,32].

Salinity induces the accumulation of reactive oxygen species (ROS) in the cells at the subcellular level, particularly in chloroplasts and mitochondria, important sources of ROS in plants subjected to salt stress, which can affect the photosynthetic process, resulting in growth inhibition and a low grain yield [55]. Plants have antioxidant mechanisms, such as CAT, POD, and PPO, for scavenging the excess ROS, which prevents cellular damage. Plant genes encode different ROS-detoxifying and ROS-producing enzymes, which aid in ROS-scavenging in the cell [56]. We found that salt tolerance correlated with higher levels of certain antioxidant enzymes. Conversely, salt-sensitive genotypes showed ineffective responses or a decline in antioxidant levels, with lower antioxidant levels than salt-tolerant genotypes, which is consistent with Acosta-Motos et al. [9]. 

The highly significant differences between the 18 genotypes used in our study indicate the existence of variance due to the relative change between the control and salinity treatments for all measured traits (Table 2). Heritability information, detailing the amount of genetic and environmental variation [57], may be relied upon to predict the reliability of the phenotypic value as an indicator of the breeding value [58]. However, heritability alone is insufficient for selection without also relying on the genetic gain (GG). A combination of both a h^2^ < 60 and GG > 30 for all traits, shows that the variation in these traits is mainly due to genetic contributions, meaning they could be drawn upon during the selection process and suggesting that the majority of the gene effects are additive (Table 2). Therefore, we can use the traits with a high h^2^ and GG when selecting for salinity tolerance [6]. Given the weakness of the predicted response for the selection of salinity tolerance based on the SDM alone, the identification of the relationships of the SDM with the studied traits could provide insight into the studied traits, which could be used indirectly to select for/enhance the SDM. In addition, understanding how the studied traits impact on the SDM (either positively or negatively) would help plant breeders in making the appropriate decisions for the breeding strategy, which is highly desirable if methods that are inexpensive, quick, and easily measurable became available [17].

In optimal conditions, the plants maintain photosynthesis at an optimal level through a balance between the size of the assimilation surface, transpiration, and chlorophyll. However, in stress conditions, plants reduce in growth due to a reduction in the number and area of the leaves. Our findings, presented in Table 2, indicate a sharp reduction in the SDM accompanied by a relative diminution of about 30% in the salt-sensitive genotypes, such as the sensitive check cultivar (Gemmeiza-9) and some DHLs that showed sensitivity, including DHL3, DHL7, DHL14, DHL15, DHL22, and DHL29. In contrast, in the salt-tolerant genotypes, such as the tolerant check cultivar (Sakha-93) and some DHLs that showed tolerance, including DHL2, DHL5, DHL21, DHL25, and DHL26, SDM reduction was considerably lower than in the salt-sensitive genotypes.

Simple correlations, without the consideration of the interactions between the SDM and related traits, may mislead plant breeders from achieving their primary objective [59]. Therefore, we used multivariate analyses (best model regression and stepwise regression). These methods were used by [60,61,62] in several studies, using all traits as independent variables so that the dependent variable allowed us to identify the relationships among traits that described salinity tolerance. Instead of considering only the SDM, other parameters, such as the RDM and RT, could be unbiased parameters and impact on the assessment of salinity tolerance (*p* > 0.01, Table 3). The stepwise regression model involves integrating as few variables as possible, because each irrelevant regressor decreases the precision of the estimated coefficients and predicted values. In addition, the presence of extra variables increases the complexity of the data collection and model maintenance. The stepwise regression model has a significant coefficient of determination (R^2^) of 0.907 from 1.000. In this study, the two independent variables, RDM and RT, could be unbiased parameters for assessing the salinity tolerance, given their contribution in the production of the SDM as a dependent variable. The great contribution of the RT on the SDM supported its significance as a selective standard in wheat. Therefore, it may be used as a criterion in screening and selection to measure the optimum genotypes for salt tolerance after removing the RDM trait due to its weak contribution, measurement difficulties, and high cost [19,63,64].

The genotypes showed natural genetic differences among themselves, given that genotypes that excel in one trait will simultaneously be inferior in other traits [15]. A dendrogram was constructed, based on the relative change of the RT and SDM, for the classification of the 18 genotypes into three main clusters. The separation and classification according to the salinity tolerance between genotypes was clear. The highest degree of salinity tolerance was in five genotypes, including DHL2, DHL5, DHL21, DHL25, and Sakha-93 (group T, Figure 1). The high value for the relative change of the RT indicates that the photosynthetic ability of the sensitive plants under salt stress were limited due to chlorine poisoning and a reduction in the shoot growth [10,65,66]. The relative change was the highest with RT and the lowest with SDM in group T (DHL2, DHL5, DHL25, DHL21, and Sakha-93) compared to group S (DHL3, DHL7, DHL8, DHL14, DHL15, DHL22, DHL29, and Gemmeiza-9), which showed quite the reverse along the line.

RT integrates the leaf water potential with the effects of osmotic adjustment (a robust mechanism to maintain cellular hydration) as a barometer of plant water status. The tolerant genotype has the power to minimize stress through the conservation of turgid leaves under stress conditions, which will have physiological advantages, such as growth and stomatal activity, and will protect and maintain the photosystem complex [67]. The relative difference in the water content of the leaf samples provides a quantitative measure of their infield hydration status. Trials can be rapidly screened for genotypes that maintain high leaf RT values during water deficit stress and vice-versa. Group T was less affected by salinity due to catalase activity, which can detoxify due to amendments in the leaf morphology, chlorophyll composition, and biochemical actives that inhibit the oxidative damage through photosynthesis, heat fragmentation by the xanthophyll pigments, and electron transfer to oxygen acceptors other than water [10,68]. The suggestion was also made that differences in the antioxidant activity between genotypes may be because of differences in the closure level of the stomata and in other responses that increase CO_2_ fixation [10]. 

### 4.2. Genetic Diversity

Another important conclusion in this study is the identification of information concerning genetic diversity. The degree of genetic variation between and within groups is the outcome of various factors, such as gene flow, hybridization, the selection effect, genetic drift, and natural and/or artificial mutagens [69]. Knowing more about the degree of molecular variation, as well as the genetic structure of genotypes, is an important tool for its maintenance [70]. Molecular markers play a key role in identifying the genetic variation of different species and/or varieties, and many studies searching for genetic diversity within wheat genotypes based on agro-morphological descriptions noted large phenotypic variation within genotypes [70,71,72]. In our study, the morphological description of genotypes was based on their salinity tolerance and showed the diversity between groups. However, morphological descriptions are not always sufficient to conclude the presence of genetic variability, owing to their vulnerability to environmental factors. DNA markers have been proven to be a reliable and precise method to detect genetic diversity, which worked to increase the chances of survival in a saline environment.

SSRs are powerful markers used in assessing polymorphisms and the degree of genetic variation of many plant species, including wheat [70]. In this study, 43 different SSR markers were tested and selected for their association with salt tolerance genes in wheat [29,32,44]. Here, we evaluated 23 SSR markers that generated polymorphisms to decipher their discriminating capacity between the salt-tolerant genotypes through identifying allele markers that are associated with each genotype. High parameter values (TNB, NPB, PPB%, PIC, and DP%) were registered, which showed that the used SSR markers are helpful in detecting the genetic variability of wheat genotypes (Table 4). We obtained 42 alleles in total, with an average of 2.33 alleles and a PIC value greater than 0.50, indicating the ability of these markers to analyze the genetic variability of wheat genotypes, which were consistent with the results obtained in wheat genotypes using different SSR markers by [30,32,44]. These findings suggest that the tested markers are informative and capable of detecting salt-tolerant genotypes.

In our results, the salt-intermediate group (I) showed the highest genetic variability (H = 0.34; I = 0.50; PPL = 76.05%), indicating more diversity among themselves compared to the salt-sensitive group (S), which showed the lowest variability (H = 0.25; I = 0.39; PPL = 66.67%) (Table 6). The higher degree of genetic diversity between genotypes and groups in our study may be attributed to the genotypes being either tolerant or sensitive towards salinity and a higher number of alleles associated with salt tolerance genes in wheat being located using the SSR markers. 

The genetic structure analysis results showed that all genotypes were assigned to three groups or clusters based on their salinity response, as indicated by the groups in Figure 1. The tolerant group (T) is detached from the other two groups and includes four DHLs from individuals of the intermediate group (I), which showed some deviation. This explains that it is possible that the morphological distance is not the principal factor in partitioning groups’ “genetic structures”, owing their effect to the environmental conditions or because salt tolerance mechanisms are polygenic in nature. Thus, it is possible that the markers we used were too few in number. The third cluster was composed entirely of individuals belonging to the salt-sensitive group (S). Many reports have revealed that the clustering of genotypes based on different agronomic, morphological, and/or physiological parameters have a high correlation with the analysis based on SSR marker data [32,73,74]. However, this is different to that previously described by other studies, including [12,75,76]. A potential explanation for this is a difference in the genotypes used and their places of origin, as well as a difference in the markers used and their types.

A comparison of genetic and morphological distances using the Mantel test is one of the most popular approaches to determine the relationship between the two. According to the Mantel test, the clustering pattern in this study is caused by both the genetic and morphological distances, as a significant positive correlation was observed (r = 0.514, *p* < 0.0001, alpha = 0.05). In addition, the SSR markers were successfully used for gene tagging or used in marker-assisted selection for agronomic traits [44,77]. 

Given the importance of the markers associated with the parameters and their contribution to the tolerance, we found 17 markers associated with the phenotypic trait (R^2^ ranged from 0.077 to 0.832), which could be considered as important indicators for describing the genetic diversity of salt tolerance in wheat. Given the importance of the relative change of the SDM and RT for the clustering of the phenotypic data, we found the cumulative coefficient of determination of the markers were powerful, being 0.853 and 0.826, respectively. Furthermore, the Xgwm 312 marker, which is used to detect the Nax1gene and seeks for Na exclusion as described by [33,34,78], was significantly correlated with the relative change of the SDM and RT (R^2^ were 0.749 and 0.628, respectively; Table 7). This was a result of the association with DNA fragments that are closely linked to specific genes affecting salt tolerance in wheat. These results confirm that the exclusion of toxic ions (Na and/or Cl^−^) from the plant cells could be used to avoid the negative effects of these ions on plant physiological processes. 

As genotyping by sequencing has become increasingly more accessible, it is likely to increase the accuracy of genetic differentiation, which assists in achieving genomic selection and/or the development of markers related to the physiological parameters of salinity tolerance [12]. The genotypes DHL2, DHL5, DHL21, DHL25, and Sakha-93 showed competitiveness, good performance, good biomass production, and high production under salt stress. Biomass production is an important indicator for evaluating salt tolerance, because it allows a direct assessment of the economic return under salt stress [32]. Therefore, the use of tolerant genotypes will enhance the possibility of introducing new salt-tolerant genotypes of wheat through selection and hybridization of the desired genotypes, which in turn will broaden the genetic base for salt tolerance breeding. 

## 5. Conclusions

Our results confirm the importance of the RT and SDM, based on the relative change in the classification of DHLs for salt tolerance compared to check cultivars. Moreover, a strong significant correlation between the morphological and genetic distances (r = 0.514, *p* < 0.0001) was observed based on the Mantel test. In total, 17 markers showed linkages with all examined parameters (R^2^ ranged from 0.077 to 0.832), and thus, they are extremely useful in detecting tolerant and sensitive genotypes to determine their usefulness for salt tolerance through marker-assisted selection.

## Figures and Tables

**Table 1:** Mean values, range of mean, coefficient of variation (CV), and LSD for the 13 measured traits of wheat genotypes grown in control and salinity conditions.

Traits	Treatments	Check Cultivars			DHLs		CV%	LSD_0.05_	LSD_0.01_
		Gemmeiza9	Giza168	Sakha93	means	Range			
RN	Control	4.52	4.18	4.66	4.40	3.34–5.33	14.54	0.53	0.70
	Salinity	5.80	5.19	5.00	5.47	4.01–6.00	10.62		
RL	Control	12.40	11.81	8.65	2.11	9.76–16.72	15.49	1.52	2.01
	Salinity	7.86	7.88	7.96	8.23	7.00–10.96	11.74		
RDM	Control	0.055	0.052	0.045	0.062	0.051–0.062	10.53	0.004	0.005
	Salinity	0.036	0.040	0.040	0.043	0.032–0.051	14.29		
SL	Control	28.33	29.17	27.07	8.73	22.40–33.60	10.41	2.12	2.82
	Salinity	20.40	22.40	22.87	21.96	17.73–28.00	11.72		
SDM	Control	0.0381	0.0351	0.0322	0.042	0.032–0.052	14.72	0.003	0.004
	Salinity	0.0251	0.0331	0.0319	0.040	0.031–0.050	17.64		
MSI	Control	79.29	79.29	75.80	79.50	71.70–86.87	5.14	7.26	9.63
	Salinity	46.38	58.15	71.62	56.97	47.97–68.41	13.29		
CHL	Control	743.76	715.43	854.06	751.32	590.75–922.01	11.00	62.95	83.58
	Salinity	671.33	591.99	849.72	683.59	549.99–834.99	13.34		
RWC	Control	92.00	94.68	98.32	93.48	90.42–95.75	2.12	2.41	3.20
	Salinity	90.46	91.76	91.97	90.66	88.44–92.80	1.22		
RT	Control	94.57	97.13	97.61	95.08	91.67–98.96	2.12	3.52	4.68
	Salinity	91.60	94.09	80.22	87.85	75.27–96.52	6.71		
WD	Control	5.43	2.87	2.39	4.92	1.04–8.33	43.07	3.52	4.68
	Salinity	8.40	5.91	19.78	12.15	3.48–24.73	49.11		
POD	Control	0.145	0.128	0.129	0.142	0.110–0.171	14.16	0.022	0.029
	Salinity	0.097	0.124	0.166	0.131	0.061–0.200	30.91		
PPO	Control	0.029	0.026	0.031	0.032	0.020–0.041	23.80	0.005	0.007
	Salinity	0.024	0.029	0.039	0.031	0.021–0.042	20.52		
CAT	Control	0.067	0.063	0.059	0.069	0.051–0.081	10.40	0.007	0.010
	Salinity	0.064	0.068	0.081	0.071	0.051–0.090	15.92		

Roots number (RN), root length (RL), root dry matter (RDM), shoot length (SL), shoot dry matter (SDM), membrane stability index (MSI), chlorophyll content (CHL), relative water content (RWC), relative turgidity (RT), water deficit (WD), peroxidase (POD), polyphenol oxidase (PPO), catalase (CAT), and doubled haploid lines (DHLs). LSD value to test interaction between the genotypes and treatments.

**Table 2:** Analysis of variance for relative change of measured traits, heritability, genetic gain, genotypic and phenotypic coefficients of variability, and relative change values for traits of 18 wheat genotypes.

S.O.V. df	RN	RL	RDM	SL	SDM	MSI	CHL	RWC	RT	WD	POD	PPO	CAT
Replications 2	0.000	0.002	0.001	0.005	0.001	0.006	0.000	0.0001	0.002	0.056	0.000	0.003	0.005
Genotypes 17	0.053	0.021	0.016	0.012	0.022	0.029	0.028	0.0010	0.012	39.198	0.088	0.099	0.029
Error 34	0.006	0.004	0.003	0.002	0.003	0.005	0.001	0.0001	0.003	0.791	0.003	0.003	0.004
h2	87.90	79.91	80.75	81.99	85.01	80.97	97.52	94.01	86.96	97.98	96.10	96.64	87.73
GG	96.73	44.62	46.22	45.83	107.71	58.95	222.61	111.57	151.41	288.11	146.88	159.97	130.05
Relative change of measured traits													
Genotypes	RN	RL	RDM	SL	SDM	MSI	CHL	RWC	RT	WD	POD	PPO	CAT
DHL2	−0.094	0.251	0.224	0.179	0.023	0.176	0.013	0.064	0.138	−2.013	−0.231	−0.178	−0.244
DHL3	−0.185	0.299	0.338	0.348	0.359	0.341	0.240	0.008	0.035	−0.852	0.580	−0.036	−0.055
DHL5	−0.382	0.316	0.211	0.167	0.026	0.187	0.056	0.048	0.143	−13.63	0.069	0.148	−0.129
DHL7	−0.200	0.314	0.315	0.217	0.304	0.362	0.007	0.012	0.041	−0.760	0.156	0.008	−0.022
DHL8	−0.414	0.323	0.340	0.269	0.203	0.314	0.252	0.017	0.043	−0.626	0.416	0.214	−0.046
DHL11	−0.169	0.235	0.212	0.179	0.129	0.279	0.123	0.027	0.094	−1.215	−0.081	−0.585	−0.192
DHL12	−0.223	0.300	0.240	0.235	0.135	0.241	0.026	0.030	0.070	−2.409	−0.030	−0.362	−0.107
DHL14	−0.455	0.334	0.315	0.279	0.353	0.417	0.141	0.017	0.013	−0.196	0.607	0.189	0.081
DHL15	−0.499	0.480	0.371	0.331	0.336	0.425	0.009	0.031	0.027	−0.603	0.113	0.360	−0.065
DHL21	−0.128	0.257	0.146	0.200	0.019	0.176	0.021	0.057	0.210	−4.183	−0.251	−0.291	−0.252
DHL22	−0.125	0.382	0.352	0.304	0.316	0.320	0.064	0.026	0.034	−0.657	0.266	−0.010	0.151
DHL23	−0.414	0.363	0.223	0.228	0.157	0.249	0.295	0.031	0.002	−0.072	0.104	−0.005	−0.057
DHL25	−0.269	0.248	0.213	0.177	0.059	0.195	0.013	0.035	0.160	−8.730	−0.276	−0.493	−0.218
DHL26	−0.165	0.254	0.258	0.125	0.084	0.252	0.010	0.039	0.100	−1.096	−0.228	−0.539	−0.076
DHL29	−0.125	0.371	0.325	0.274	0.318	0.303	0.013	0.006	0.026	−0.343	0.095	0.097	0.241
Sakha93	−0.074	0.080	0.120	0.155	0.009	0.055	0.005	0.065	0.178	−7.282	−0.286	−0.263	−0.379
Giza168	−0.241	0.333	0.231	0.232	0.144	0.267	0.173	0.031	0.031	−1.056	0.031	−0.132	−0.080
Gemmeiza9	−0.283	0.366	0.336	0.280	0.348	0.415	0.097	0.017	0.031	−0.547	0.335	0.154	0.049
LSD 0.05	0.132	0.105	0.092	0.076	0.093	0.121	0.043	0.047	0.066	1.457	0.096	0.094	0.097
LSD 0.01	0.175	0.140	0.122	0.101	0.124	0.161	0.057	0.063	0.087	1.940	0.128	0.126	0.129

Roots number (RN), root length (RL), root dry matter (RDM), shoot length (SL), shoot dry matter (SDM), membrane stability index (MSI), chlorophyll content (CHL), relative water content (RWC), relative turgidity (RT), water deficit (WD), peroxidase (POD), polyphenol oxidase (PPO), catalase (CAT), broad sense heritability (h2, %), Genetic gain (GG %), and doubled haploid line (DHL).

**Table 3:** Best model and stepwise regression for shoot dry matter (dependent variable) with 12 measured traits (independent variables).

Source	Best Model Regression			Stepwise Regression			
	Regression Coefficient	*P*-value	R^2^ Com.	Regression Coefficient	*P*-value	R^2^ Par.	R^2^ Com.
Intercept	−0.122	0.111		−0.087	0.365		
RN	0.281	**0.017**		0.000			
RL	−0.334	0.070		0.000			
SL	0.440	**0.047**		0.000			
MSI	0.746	**0.003**		0.000			
CHL	0.000			0.000			
POD	0.000			0.000			
PPO	−0.074	0.094		0.000			
CAT	−0.195	0.079		0.000			
WD	−0.017	**0.001**		0.000			
RWC	0.000			0.000			
RDM	0.569	**0.042**		0.928	**0.004**	0.072	0.072
RT	−2.322	**< 0.0001**		−1.392	**0.000**	0.835	0.907
RN/RL/SL/MSI/PPO/CAT/WD/RDM/RT			0.988				
Residual			0.022				0.093

Roots number (RN), root length (RL), root dry matter (RDM), shoot length (SL), membrane stability index (MSI), chlorophyll content (CHL), relative water content (RWC), relative turgidity (RT), water deficit (WD), peroxidase (POD), polyphenol oxidase (PPO), catalase (CAT), coefficient partial determination (R^2^ Par), and cumulative coefficient determination (R^2^ Com.).

**Table 4:** List of 23 simple sequence repeat (SSR) makers, total number of bands, number of polymorphic bands, percentage of polymorphic bands, polymorphism information content, and discrimination power for eighteen wheat genotypes.

Makers	TNB	NPB	PPB%	PIC	DP %	No. of Bands in Each Group		
						T	I	S
Barc182	2	1	50%	0.31	2.86	2/2	2/2	2/2
Cfd1	2	1	50%	0.66	2.86	2/2	2/2	2/2
Cfd18	1	1	100%	0.85	2.86	1/1	1/1	1/1
Cfd60	2	2	100%	0.96	5.71	2/2	2/2	0/2
Cfd66	2	1	50%	0.77	2.86	2/2	2/2	2/2
Gwm148	3	3	100%	0.95	8.57	3/3	2/3	2/3
Gwm174	1	1	100%	0.63	2.86	1/1	1/1	1/1
Gwm205	3	3	100%	0.83	8.57	3/3	2/3	0/3
Gwm210	2	2	100%	0.93	5.71	2/2	2/2	0/2
Gwm247	1	1	100%	0.63	2.86	1/1	1/1	1/1
Gwm249	2	2	100%	0.66	5.71	2/2	2/2	2/2
Gwm299	2	1	50%	0.80	2.86	2/2	2/2	2/2
Gwm312	2	2	100%	0.83	5.71	2/2	2/2	1/2
Gwm314	1	1	100%	0.69	2.86	1/1	1/1	1/1
Gwm340	2	1	50%	0.69	2.86	2/2	2/2	2/2
Gwm350	3	2	67%	0.69	5.71	3/3	3/3	3/3
Gwm455	1	1	100%	0.80	2.86	1/1	1/1	1/1
Gwm614	1	1	100%	0.83	2.86	1/1	1/1	0/1
Wmc11	3	2	67%	0.40	5.71	3/3	3/3	3/3
Wmc503	2	2	100%	0.92	5.71	2/2	2/2	1/2
Wmc661	2	2	100%	0.82	5.71	2/2	2/2	2/2
Wmc170	1	1	100%	0.85	2.86	1/1	0/1	0/1
Wmc18	1	1	100%	0.92	2.86	0/1	1/1	1/1

TNB: total number of bands, NPB: number of polymorphic bands, PPB%: percentage of polymorphic bands, PIC: polymorphism information content, Dp: discrimination power, T: tolerant, I: intermediate, and S: sensitive.

**Table 5:** Summary of analysis of molecular variance (AMOVA).

Source		df	SS	MS	Est. Variance	% Variance
Among groups (AG)		2	26.546	13.273	1.812	20%
Within groups (WG)		15	126.676	8.445	7.445	80%
Total		17	153.222		9.257	100%
PhiPT (value/P<)	0.196					
P (rand perm.999)	0.001					

Populations refer to the wheat clusters (T, I, and S) in Figure 1. Df, degree of freedom; SS, sum of squares; MS, mean square; Est. Variance, estimated variance; % Variance, percent variance; PhiPT, estimate of genetic distance among groups; P(randperm.999), significance of genetic distance at 999 random permutations; and PhiRT = AG/(AG + WG) = AG/TOT.

**Table 6:** Intra-groups diversity measures of wheat genotypes based on SSR data.

Groups Cluster	T	I	S	Total
Sample size	5	6	7	18
Mean No. of different alleles	1.81 ± 0.08	1.81 ± 0.08	1.69 ± 0.11	1.87 ± 0.05
Mean No. of effective alleles = 1/(p^2^ + q^2^)	1.57 ± 0.05	1.61 ± 0.06	1.41 ± 0.05	1.63 ± 0.03
Shannon’s information index (I) = −1 × (p × Ln (p) + q * Ln(q))	0.48 ±0.04	0.50 ± 0.04	0.39 ± 0.04	0.56 ± 0.02
Mean expected heterozygosity = 2 × p × q	0.32 ± 0.03	0.34± 0.03	0.25 ± 0.03	0.37 ± 0.02
Unbiased expected heterozygosity = (2N/(2N−1)) × He	0.36 ± 0.03	0.37 ± 0.03	0.27 ± 0.03	0.40 ± 0.02
Nei’s gene diversity (H) = 1- (p^2^ + q^2^)	0.33 ± 0.17	0.34 ± 0.18	0.25 ± 0.16	0.37 ± 0.17
Number of polymorphic loci	33	32	28	35
Percentage of polymorphic loci (PPL)	78.14%	76.05%	66.67%	83.33%
No. of bands unique to a single group	2	0	0	35
Genetic distance of Nei (1978) among salinity groups of DHLs wheat				
	T	I	S	
	0.000			T
	0.089	0.000		I
	0.138	0.116	0.000	S

T: Tolerant; I: Intermediate; and S: Sensitive.

**Table 7:** Selection of the influential markers (independent variables) with 13 studied traits (dependent variable) for both treatments of control and salinity and relative change based on stepwise multiple linear regression (SMLR) analysis.

Traits	Treatments	Markers	R^2^ Par	R^2^ Com.	P-value*
RN	Salinity	Cfd60	0.389	-	0.015
		Wmc18	0.168	0.557	0.031
	Relative change	Wmc502	0.282	-	0.013
		Wmc11	0.225	0.507	0.019
RL	Control	Gwm249	0.345	-	0.012
		Barc182	0.165	0.510	0.040
	Salinity	Cfd60	0.524	-	0.038
		Gwm614	0.123	0.647	0.00018
	Relative change	Gwm241	0.501	-	0.002
		Gwm249	0.238	0.739	0.00119
RDM	Control	Gwm335	0.272	0.272	0.027
	Salinity	Gwm241	0.435	0.435	0.003
	Relative change	Wmc18	0.576	-	0.007
		Cfd66	0.168	0.744	< 0.0001
SL	Control	Wmc60	0.458	-	0.020
		Wmc502	0.168	0.626	0.003
	Salinity	Barc182	0.334	-	0.024
		Wmc502	0.198	0.532	0.004
	Relative change	Gwm241	0.679	-	< 0.0001
		Wmc60	0.077	0.756	0.046
SDM	Control	Wmc502	0.259	-	0.003
		Gwm614	0.239	0.498	0.017
	Salinity	Gwm312	0.539	-	0.009
		Wmc502	0.174	0.714	0.0001
	Relative change	Gwm145	0.749	-	0.005
		Gwm614	0.104	0.853	< 0.0001
MSI	Salinity	Gwm160	0.685	-	< 0.0001
		Cfd66	0.085	0.769	0.0332
	Relative change	Gwm160	0.595	-	0.018
		Gwm340	0.129	0.724	< 0.0001
CHL	Control	Wmc18	0.239	0.239	0.003
	Salinity	Gwm340	0.380	-	0.003
		Cfd13	0.189	0.570	0.0213
	Relative change	Gwm155	0.249	-	0.010
		Gwm145	0.257	0.506	0.014
POD	Control	Cfd13	0.689	-	< 0.0001
		Cfd60	0.093	0.782	0.023
	Salinity	Gwm241	0.586	-	0.026
		Gwm614	0.120	0.706	0.002
	Relative change	Gwm241	0.576	-	0.024
		Gwm335	0.125	0.702	< 0.0001
PPO	Control	Wmc502	0.418	-	0.002
		Wmc11	0.162	0.580	0.030
	Salinity	Gwm335	0.595	-	0.0001
		Gwm312	0.176	0.771	0.004
	Relative change	Gwm335	0.604	0.604	0.0001
CAT	Control	Wmc11	0.241	-	0.0333
		Gwm249	0.203	0.444	0.011
	Salinity	Gwm241	0.726	-	0.0002
		Gwm614	0.091	-	< 0.0001
		Gwm155	0.114	0.931	0.0003
	Relative change	Gwm241	0.542	-	0.0101
		Gwm335	0.167	0.710	< 0.0001
RWC	Control	Cfd60	0.224	-	0.0037
		Cfd66	0.247	0.471	0.018
	Salinity	Gwm241	0.832	-	0.0060
		Gwm340	0.068	0.900	< 0.0001
	Relative change	Gwm241	0.808	-	0.0019
		Gwm340	0.093	0.902	< 0.0001
WD	Control	Cfd60	0.224	-	0.0037
		Cfd66	0.247	0.471	0.018
	Salinity	Gwm241	0.832	-	0.0060
		Gwm340	0.068	0.900	< 0.0001
	Relative change	Gwm241	0.413	-	0.0004
		Cfd66	0.162	0.574	0.031
RT	Control	Gwm145	0.514	-	0.0051
		Gwm614	0.203	0.717	0.002
	Salinity	Wmc502	0.215		0.0249
		Gwm312	0.185	0.400	0.048
	Relative change	Gwm312	0.628		0.0009
		Gwm614	0.198	0.826	0.0002

Roots number (RN), root length (RL), root dry matter (RDM), shoot length (SL), shoot dry matter (SDM), membrane stability index (MSI), chlorophyll content (CHL), coefficient partial determination (R^2^_Par_)_,_ and cumulative coefficient determination (R^2^_Com._). * means *P*-value of coefficient partial determination. Relative water content (RWC), relative turgidity (RT), water deficit (WD), peroxidase (POD), polyphenol oxidase (PPO), catalase (CAT), coefficient partial determination (R^2^ Par), and cumulative coefficient determination (R^2^ Com.). * means P-value of coefficient partial determination.

## Supplementary Materials

The following are available online at https://www.mdpi.com/2223-7747/9/3/287/s1. Figure S1. Mean performance of chlorophyll content (ChL) and membrane stability index (MSI) for eighteen wheat genotypes grown under control and salinity conditions. Table S1. The pedigree and salt tolerance of the three checked bread wheat cultivars used in this study. Table S2. List of SSR markers used in the study across 18 wheat genotypes. Table S3. Analysis of the differences between the groups of relative turgidity shoot dry matter and traits. Table S4. Morphological distance (MD) estimation between 18 wheat genotypes based on the relative change of the RT and SDM. Table S5. Genetic distance (GD) estimation between 18 wheat genotypes using SSR molecular markers.
