# Supplementary material for: Morphological and Genetic Diversity within Salt Tolerance Detection in Eighteen Wheat Genotypes
Source: Plants (Basel). 2020 Feb 25;9(3):287. doi: 10.3390/plants9030287 (PMC7154827; doi:10.3390/plants9030287)
Supplement: Supplementary file 1 [file plants-09-00287-s001.pdf]

Figure 1. Mean performance of chlorophyll content (ChL) and membrane stability index (MSI) for eighteen wheat genotypes grown under control and salinity conditions.

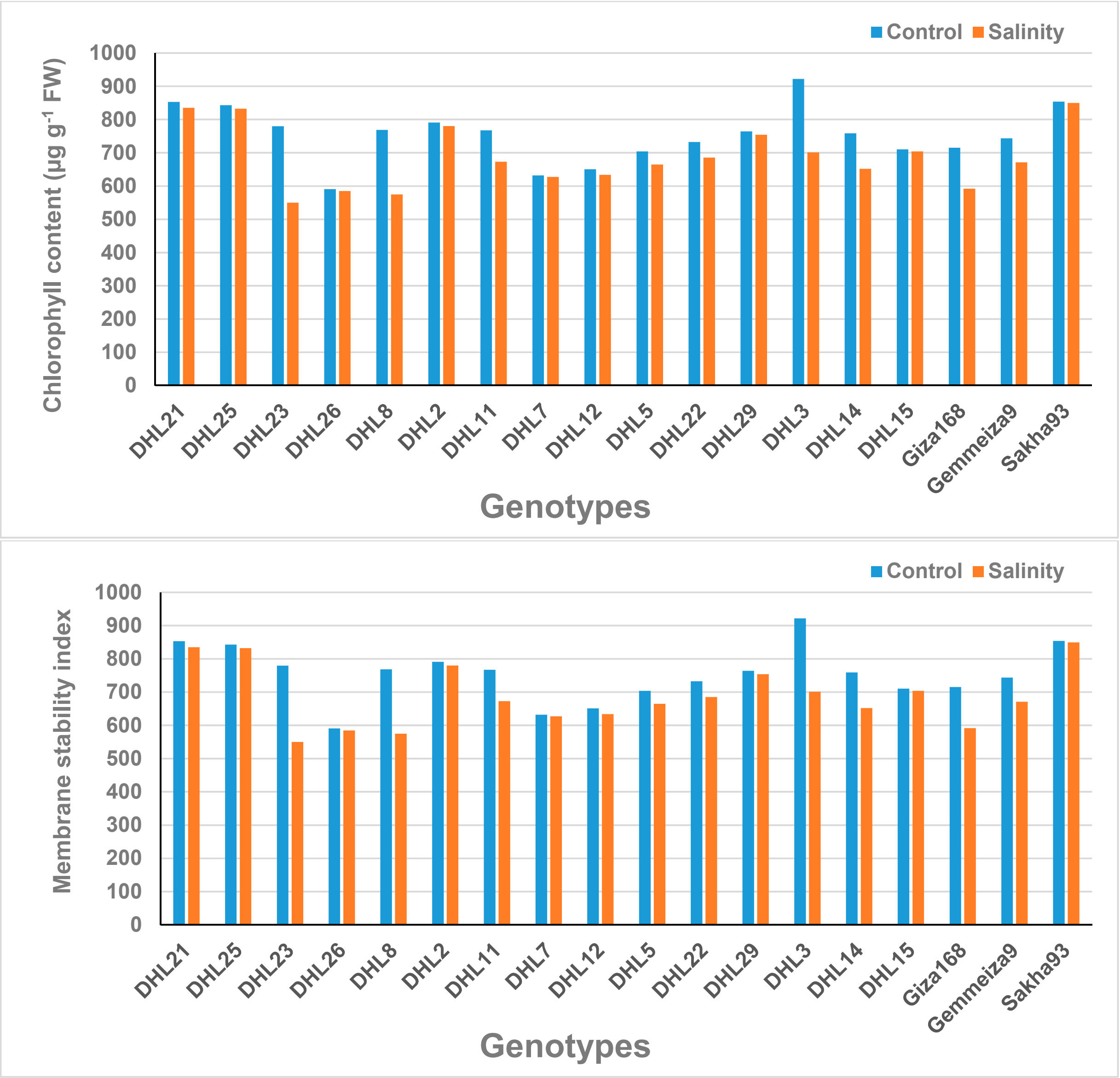

Table S1. The pedigree and salt tolerance of the three check bread wheat cultivars used in this study.

| Genotypes | Pedigree | Salt tolerance |
|---|---|---|
| Check cultivars | | |
| Sakha-93 | Sakha 92/TR810328 S8871-IS-2S-IS-0S | Tolerant |
| Giza-168 | MRL/BUC//SERICM 93046-8 M-OY-OM-2Y-OB-OGZ. | Moderate |
| Gemmeiza-9 | Ald"s"/Huac//CMH74 .630/SxCGM 4583 -5GM- 1GM- OGM | Sensitive |
| Doubled haploid line (DHLs) | | |
| 15 DHLs | The DHLs were obtained from the Agronomy Department, Faculty of Agriculture, Al-Azhar University, Nasr City, Cairo, Egypt, and published by El-Hennawy et al. (2011), and selected based on their good grain yield performance as follows:<br>DHLs (21,22,23,25), derived from the cross (Line-115 × Gemmeiza-7)<br>DHLs (5,7,8,11), derived from the cross (Line-115 × Giza-164) | |

Table S2. List of SSR markers used in the study across 18 wheat genotypes.

| Oligo Name | Chromosome | Sequence 5 to 3 | | Annealing temp. | Repeat |
|---|---|---|---|---|---|
| | | F | R | | |
| cslinkkna2 | 5A | TCTCCATCATTCAACATCAATCG | TGTAGCTCGTCGGGGTGTGTTGC | 58 | - |
| Xbarc182 | 7B | CCATGGCCAACAGCTCAAGGTCTC | CGCAAAACCGCATCAGGGAAGCACCAAT | 58 | (CT)15 |
| Xcfd1 | 6B,6D,6A | ACCAAAGAACTTGCCTGGTG | AAGCCTGACCTAGCCCAAAT | 60 | (GCC)6 |
| Xcfd13 | 6B,6D | CCACTAACCAAGCTGCCATT | TTTTTGGCATTGATCTGCTG | 60 | (CT)20(TGTA)3 |
| Xcfd18 | 5D | CATCCAACAGCACCAAGAGA | GCTACTACTATTTCATTGCGACCA | 60 | (GA)25 |
| Xcfd183 | 5D | ACTTGCACTTGCTATACTTACGAA | GTGTGTCGGTGTGTGGAAAG | 60 | (CA)22 |
| Xcfd19 | 1,5,6D | TACGCAGGTTTGCTGCTTCT | GGAGTTCACAAGCATGGGTT | 60 | (GA)18 |
| Xcfd46 | 7D | TGGTGGTATAGTCGTTGGAGC | CCACACACACACACCATCAA | 60 | (GT)29 |
| Xcfd49 | 6D | TGAGTTCTTCTGGTGAGGCA | GAATCGGTTCACAAGGGAAA | 60 | (GA)33 |
| Xcfd60 | 6D,5B | TGACCGGCATTCAGTATCAA | TGGTCACTTTGATGAGCAGG | 60 | (CA)25 |
| Xcfd66 | 7D | AGGTCTTGGTGGTTTTGGTG | TTTTCACATGCCCACAGTTG | 60 | (GC)9(AG)60 |
| Xcfd9 | 3D | TTGCACGCACCTAAACTCTG | CAAGTGTGAGCGTCGG | 60 | (TC)29 |
| Xgwm133 | 3A,6,7B,6D | ATCTAAACAAGACGGCGGTG | ATCTGTGACAACCGGTGAGA | 60 | (CT)39imp |
| Xgwm148 | 2B | GTGAGGCAGCAAGAGAGAAA | CAAAGCTTGACTCAGACCAAA | 60 | (CA)22 |
| Xgwm174 | 5D | GGGTTCCTATCTGGTAAATCCC | GACACACATGTTCCTGCCAC | 55 | (CT)22 |
| Xgwm181 | 3B | TCA TTG GTA ATG AGG AGA GA | GAA CCA TTC ATG TGC ATG TC | 51 | (GA)28 |
| Xgwm205 | 5D,5A | CGACCCGGTTCACTTCAG | AGTCGCCGTTGTATAGTGCC | 60 | (CT)21 |
| Xgwm210 | 2A | TGCATCAAGAATAGTGTGGAAG | TGAGAGGAAGGCTCACACCT | 55 | (GA)20 |
| Xgwm247 | 3A,3B | GCAATCTTTTTTCTGACCACG | ATG TGC ATG TCG GAC GC | 55 | (GA)24 |
| Xgwm249 | 2D | CAAATGGATCGAGAAAGGGA | CTGCCATTTTTCTGGATCTACC | 55 | (GA)11(GGA)8 |
| Xgwm296 | 2D,7D | AATTCAACCTACCAATCTCTG | GCCTAATAAACTGAAAACGAG | 55 | (CT)28 |
| Xgwm299 | 3B,2B | ACTACTTAGGCCTCCCGCC | TGACCCACTTGCAATTCATC | 55 | (GA)31(TAG)4 |
| Xgwm312 | 2A | ATCGCATGATGCACGTAGAG | ACATGCATGCCTACCTAATGG | 60 | (GA)37 |
| Xgwm314 | 3D,4B | AGGAGCTCCTCTGTGCCAC | TTCGGGACTCTCTTCCCTG | 55 | (CT)25imp |
| Xgwm335 | 5B | CGTACTCCACTCCACACGG | CGGTCCAAGTGCTACCTTTC | 55 | (GA)14(GCGT)3 |
| Xgwm340 | 3B | GCAATCTTTTTTCTGACCACG | ACGAGGCAAGAACACACATG | 60 | (GA)26 |
| Xgwm350 | 7D | ACCTCATCCACATGTTCTACG | GCATGGATAGGACGCCC | 55 | (GT)14 |
| Xgwm413 | 1B,1A | TGCTTGTCTAGATTGCTTGGG | GATCGTCTCGTCCTTGGCA | 60 | (GA)18 |
| Xgwm455 | 2D,6B | ATTCGGTTCGCTAGCTACCA | ACGGAGAGCAACCTGCC | 55 | (GT)19imp |

| | | | | | |
|---|---|---|---|---|---|
| Xgwm539 | 2D | CTGCTCTAAGATTCATGCAACC | GAGGCTTGTGCCCTCTGTAG | 60 | (GA)27 |
| Xgwm614 | 2B,2A,2D | GATCACATGCATGCGTCATG | TTTTACCGTTCCGGCCTT | 60 | (GA)23imp |
| Xgwm674 | 3A | TCGAGCGATTTTTCCTGC | TGACCGAGTTGACCAAAACA | 60 | (CT)16CCC(GT)4 |
| Xwmc11 | 3A, 3D | TTGTGATCCTGGTTGTGTTGTGA | CACCCAGCCGTTATATATGTTGA | 61 | - |
| Xwmc169 | 3, 5A | TACCCGAATCTGGAAAATCAAT | TGGAAGCTTGCTAACTTTGGAG | 61 | (CA)25 65 to 114 |
| Xwmc17 | 7A, 7B | ACCTGCAAGAAATTAGGAACTC | CTAGTGTTTCAAATATGTCGGA | 51 | - |
| Xwmc170 | 2A,2D | ACATCCACGTTTATGTTGTTGC | TTGGTTGCTCAACGTTTACTTC | 61 | (CA)19 378 to 415 |
| Xwmc18 | 2D | CTGGGGCTTGGATCACGTCATT | AGCCATGGACATGGTGTCCTTC | 61 | (CA)(CT) CM* |
| Xwmc367 | 1B | CTGACGTTGATGGGCCACTATT | GTGGTGGAAGAGGAAGGAGAGG | 61 | (GCC)5 125 to 139 |
| Xwmc419 | 4B | GTTTCGGATAAAACCGGAGTGC | ACTACTTGTGGGTTATCACCAGCC | 61 | (GA)16 111 to 142 |
| Xwmc432 | 1D | ATGACACCAGATCTAGCAC | AATATTGGCATGATTACACA | 51 | (GT)14 238 to 265 |
| Xwmc44 | 1B | GGTCTTCTGGGCTTTGATCCTG | TGTTGCTAGGGACCCGTAGTGG | 61 | (GT)35 341 to 410 |
| Xwmc503 | 2D | GCAATAGTTCCCGCAAGAAAAG | ATCAACTACCTCCAGATCCCGT | 61 | (GT)11 112 to 133 |
| Xwmc661 | 2B | CCACCATGGTGCTAATAGTGTC | AGCTCGTAACGTAATGCAACTG | 61 | - |

*Compound microsatellite

Table S3. Analysis of the differences between the groups of relative turgidity (RT) and shoot dry matter (SDM) traits.

| Groups | RT | | SDM | |
|---|---|---|---|---|
| I | 0.030 b | | 0.334 a | |
| S | 0.057 b | | 0.142 b | |
| T | 0.166 a | | 0.027 c | |
| Pr > F | 0.000 | | 0.000 | |
| Significant | Yes | | Yes | |
| Summary (LS means) of all pairwise comparisons for groups (Fisher, LSD) | | | | |
| Contrast | RT | | SDM | |
| | P value | Significant | P value | Significant |
| T vs I | < 0.0001 | Yes | < 0.0001 | Yes |
| T vs S | < 0.0001 | Yes | < 0.0001 | Yes |
| S vs I | 0.0954 | No | < 0.0001 | Yes |

Tolerant (T), intermediate (I) and sensitive.

Table S4. Morphological distance estimation between 18 wheat genotypes using relative change of relative turgidity and shoot dry matter traits.

| Genotypes | DHL2 | DHL3 | DHL5 | DHL7 | DHL8 | DHL11 | DHL12 | DHL14 | DHL15 | DHL21 | DHL22 | DHL23 | DHL25 | DHL26 | DHL29 | Sakha93 | Giza168 |
|---|---|---|---|---|---|---|---|---|---|---|---|---|---|---|---|---|---|
| DHL3 | 0.352 | | | | | | | | | | | | | | | | |
| DHL5 | 0.006 | 0.350 | | | | | | | | | | | | | | | |
| DHL7 | 0.298 | 0.055 | 0.296 | | | | | | | | | | | | | | |
| DHL8 | 0.203 | 0.157 | 0.203 | 0.102 | | | | | | | | | | | | | |
| DHL11 | 0.115 | 0.237 | 0.114 | 0.183 | 0.089 | | | | | | | | | | | | |
| DHL12 | 0.131 | 0.227 | 0.131 | 0.172 | 0.073 | 0.024 | | | | | | | | | | | |
| DHL14 | 0.353 | 0.023 | 0.352 | 0.056 | 0.153 | 0.238 | 0.225 | | | | | | | | | | |
| DHL15 | 0.332 | 0.025 | 0.331 | 0.034 | 0.134 | 0.217 | 0.205 | 0.022 | | | | | | | | | |
| DHL21 | 0.072 | 0.383 | 0.067 | 0.332 | 0.248 | 0.160 | 0.182 | 0.388 | 0.366 | | | | | | | | |
| DHL22 | 0.311 | 0.043 | 0.310 | 0.014 | 0.114 | 0.196 | 0.185 | 0.042 | 0.021 | 0.345 | | | | | | | |
| DHL23 | 0.190 | 0.205 | 0.192 | 0.153 | 0.061 | 0.095 | 0.071 | 0.196 | 0.181 | 0.249 | 0.163 | | | | | | |
| DHL25 | 0.043 | 0.325 | 0.037 | 0.272 | 0.185 | 0.097 | 0.118 | 0.328 | 0.307 | 0.064 | 0.286 | 0.185 | | | | | |
| DHL26 | 0.072 | 0.282 | 0.073 | 0.228 | 0.131 | 0.045 | 0.059 | 0.282 | 0.262 | 0.128 | 0.241 | 0.121 | 0.066 | | | | |
| DHL29 | 0.315 | 0.043 | 0.314 | 0.020 | 0.116 | 0.200 | 0.188 | 0.037 | 0.018 | 0.351 | 0.008 | 0.163 | 0.291 | 0.245 | | | |
| Sakha93 | 0.042 | 0.378 | 0.039 | 0.325 | 0.236 | 0.147 | 0.166 | 0.381 | 0.360 | 0.033 | 0.339 | 0.229 | 0.053 | 0.109 | 0.344 | | |
| Giza168 | 0.161 | 0.216 | 0.162 | 0.161 | 0.060 | 0.064 | 0.040 | 0.210 | 0.192 | 0.218 | 0.173 | 0.032 | 0.154 | 0.090 | 0.174 | 0.199 | |
| Gemmeiza9 | 0.342 | 0.012 | 0.341 | 0.045 | 0.146 | 0.228 | 0.217 | 0.019 | 0.013 | 0.375 | 0.032 | 0.194 | 0.316 | 0.273 | 0.031 | 0.369 | 0.205 |

Doubled haploid line (DHL)

Table S5. Genetic distance estimation between 18 wheat genotypes using SSR molecular markers.

| Genotypes | DHL2 | DHL3 | DHL5 | DHL7 | DHL8 | DHL11 | DHL12 | DHL14 | DHL15 | DHL21 | DHL22 | DHL23 | DHL25 | DHL26 | DHL29 | Sakha93 | Giza168 |
|---|---|---|---|---|---|---|---|---|---|---|---|---|---|---|---|---|---|
| DHL3 | 0.731 | | | | | | | | | | | | | | | | |
| DHL5 | 0.423 | 0.667 | | | | | | | | | | | | | | | |
| DHL7 | 0.846 | 0.611 | 0.821 | | | | | | | | | | | | | | |
| DHL8 | 0.781 | 0.741 | 0.645 | 0.708 | | | | | | | | | | | | | |
| DHL11 | 0.774 | 0.731 | 0.633 | 0.846 | 0.607 | | | | | | | | | | | | |
| DHL12 | 0.773 | 0.625 | 0.696 | 1.000 | 0.833 | 0.826 | | | | | | | | | | | |
| DHL14 | 0.654 | 0.125 | 0.593 | 0.650 | 0.714 | 0.750 | 0.500 | | | | | | | | | | |
| DHL15 | 0.958 | 0.667 | 0.833 | 0.867 | 0.870 | 0.913 | 0.727 | 0.706 | | | | | | | | | |
| DHL21 | 0.700 | 0.867 | 0.645 | 0.852 | 0.750 | 0.655 | 0.783 | 0.800 | 0.960 | | | | | | | | |
| DHL22 | 0.920 | 0.625 | 0.846 | 0.417 | 0.783 | 0.826 | 0.933 | 0.667 | 0.727 | 0.880 | | | | | | | |
| DHL23 | 0.767 | 0.815 | 0.667 | 0.840 | 0.417 | 0.630 | 0.870 | 0.786 | 0.909 | 0.774 | 0.818 | | | | | | |
| DHL25 | 0.813 | 0.731 | 0.677 | 0.800 | 0.742 | 0.480 | 0.875 | 0.750 | 0.958 | 0.500 | 0.773 | 0.630 | | | | | |
| DHL26 | 0.654 | 0.750 | 0.593 | 0.826 | 0.714 | 0.409 | 0.857 | 0.769 | 0.900 | 0.667 | 0.909 | 0.786 | 0.704 | | | | |
| DHL29 | 0.714 | 0.591 | 0.655 | 0.727 | 0.577 | 0.714 | 0.750 | 0.500 | 0.789 | 0.724 | 0.684 | 0.704 | 0.714 | 0.821 | | | |
| Sakha93 | 0.647 | 0.829 | 0.639 | 0.882 | 0.730 | 0.563 | 0.793 | 0.806 | 0.900 | 0.484 | 0.939 | 0.750 | 0.563 | 0.613 | 0.743 | | |
| Giza168 | 0.692 | 0.682 | 0.679 | 0.870 | 0.793 | 0.786 | 0.647 | 0.591 | 0.765 | 0.750 | 0.789 | 0.821 | 0.867 | 0.760 | 0.667 | 0.688 | |
| Gemmeiza9 | 0.846 | 0.438 | 0.821 | 0.533 | 0.708 | 0.800 | 0.813 | 0.500 | 0.583 | 0.893 | 0.273 | 0.739 | 0.800 | 0.875 | 0.667 | 0.882 | 0.700 |

Doubled haploid line (DHL)
